# Supplementary material for: Continuum of care for maternal and newborn health services in Nepal: An analysis from demographic and health survey 2022
Source: PLoS One. 2025 Mar 24;20(3):e0319033. doi: 10.1371/journal.pone.0319033 (PMC11932462; doi:10.1371/journal.pone.0319033)
Supplement: Attachment — Submitted filename: Response to reviewers R2.docx [file pone.0319033.s004.docx]

**Response to reviewers**

**Feedback:** Rather than only including the analysis description in the methods section of the abstract, I suggest adding a sentence about the study design and sample size before discussing the analysis.

**Response**: Thank you for the feedback. We have revised as suggested

**Feedback:** In the list of independent variables, I recommend clarifying media exposure by specifying examples like newspapers, TV, and radio. This will enhance readers' understanding.

**Response**: Thank you for the feedback. We have revised as suggested

**Feedback:** Since the authors have three outcomes, I suggest replacing 'multivariable' with 'multivariate analysis' throughout the manuscript, where applicable.

**Response**: Thank you for the feedback. Rather than having all outcome variables in single model, we ran multiple outcome variable with one outcome variable at a time. We prefer to use the term multivariable analysis. This aligns with Essential Medical Statistics by [Betty R. Kirkwood,](https://www.wiley.com/en-be/search?filters%5bauthor%5d=Betty%20R.%20Kirkwood&pq=++) [Jonathan A. C. Sterne](https://www.wiley.com/en-be/search?filters%5bauthor%5d=Jonathan%20A.%20C.%20Sterne&pq=++)

**Feedback:** I recommend not mentioning R Studio as an analysis tool, as it is an environment rather than a statistical tool. If you wish to include it, please also specify its version.

**Response**: Thank you for the feedback. We have revised as suggested

**Feedback:** I hope the authors checked for multicollinearity before conducting the regression analysis. I suggest mentioning this explicitly, as variables such as age at delivery and birth order could be correlated.

**Response**: Thank you for the feedback. We have revised as suggested

**Reviewer 2**
**Feedback:** The manuscript requires a thorough review of data consistency and comprehensive editing especially in findings and discussion to ensure it is ready for publication.

**Response**: Thank you for the feedback. We have thoroughly rechecked the manuscript for consistency of data and revised the manuscript.

**Feedback:** The first paragraph presents the magnitude of problem in global context. It would be beneficial if the authors update this with recent data to ensure that that they are discussing the current situation more accurately.

**Response**: Thank you for the feedback. We have revised as suggested

**Feedback:** Para 3: In this para, the authors have presented the leading causes of maternal mortality. For more comprehensive view, it would be better if they present the leading causes for neonatal mortality as well.

**Response**: Thank you for the feedback. We have revised as suggested

**Feedback:** This (result) section requires a thorough consistency check, as there are several issues with data consistency throughout the first paragraph and table-1. The figures reported in Table 1, (the point estimates and their respective confidence intervals), do not align in many cases for both urban and rural groups. For example, the point estimate of 15.68 (95%CI: 18.12 to 23.30) for the urban group and 20.59 (95%CI: 13.28 to 18.42) for the rural group suggest that the reported point estimate does not fall within the given confidence interval. This inconsistency should be addressed. I recommend that Table 1 undergo a thorough review and verification to make sure that all reported values are consistent and correct. Although the authors have stated that they checked the consistency of the findings, my random check shows further discrepancies now that needs to be corrected.

**Response**: Thank you for the feedback. We apologize for inconsistency of data. We have now revised the table and write up and rechecked other tables too.

**Feedback:** There appears to be some redundancy in the background section. Please review and address this. Additionally, lines 325–328 could be consolidated to avoid repetition.

**Response**: Thank you for the feedback. We have revised as suggested

**Feedback:** Both the results and the discussion section need to be revised/edited to improve clarity. This will help the readers understand easily what the authors mean to say.

**Response**: Thank you for the feedback. We have revised as suggested

**Feedback:** Line 282-289: I recommend comparing the coverage over time using similar data sources, if available. Moreover, it would be beneficial to compare the findings with more recent data if available. For example, instead of comparing with the 2017-18 DHS data, the authors could consider referencing the Bangladesh Survey 2022 for a more recent statistic.

**Response**: Thank you for feedback. We have compared our findings with most recent data from DHS that have comparable methodology across different setting.
