# Supplementary material for: Continuum of care for maternal and newborn health services in Nepal: An analysis from demographic and health survey 2022
Source: PLoS One. 2025 Mar 24;20(3):e0319033. doi: 10.1371/journal.pone.0319033 (PMC11932462; doi:10.1371/journal.pone.0319033)
Supplement: Attachment — Submitted filename: Review_.docx [file pone.0319033.s001.docx]

**Comments**

The authors have conducted an analysis addressing critical issues, and addressing the gap in the continuum of care is essential for policymakers and program implementers. Once the below comments are addressed, I would recommend this manuscript for publication. However, I suggest reviewing the introduction and discussion sections with experts who have extensive knowledge about the current policies and programs related to maternal and neonatal health.

1. The authors mentioned logistic regression in the abstract, yet Poisson regression is detailed in the methodology section. Additionally, the findings present logistic regression output. Please review the report carefully to ensure coherence.
2. Be consistent in the use of decimal places, either using one or two after the decimal point throughout the document.
3. Regarding the statement, "Madhesh residents had lower odds (AOR: 0.47, 95% CI: 0.23 - 0.99), Sudurpaschim had 27 higher odds (AOR: 2.37, 95% CI: 1.17 - 4.82) for ≥4 ANC visits and health facility delivery than Koshi," I question the significance of the upper limit of 0.99. If rounded to a single decimal place, it may not appear significant. Furthermore, please confirm if the associated p-value is less than 0.05.
   It seems like the authors are trying to forcefully emphasize significance.
4. I suggest adding 95% confidence intervals (CI) in the last sentence of the methods section in the abstract. The authors overlooked mentioning CI.
5. In the introduction section of the main body, the authors discuss the SDG target for neonatal mortality. I suggest including a similar discussion for maternal mortality ratio.
6. Consider including information about the policy scenario concerning maternal and neonatal health in Nepal in the introduction section. For example, mention initiatives like the Nepal Safe Motherhood and Newborn Health Road Map 2030 and elaborate on its focus on the continuum of care.
7. It seems unnecessary for authors to use the abbreviation "MCVs" in parentheses (line # 73) since it's not used later in the text.
8. Please ensure the survey year is mentioned in the opening sentence of the methods section.
9. Simplify the discussion of sampling procedures in the methods section. Instead of detailed procedures, provide a brief overview and mention the total number of strata, refer readers to the main report for further details with citations. Additionally, consider including a flowchart illustrating the process leading to the selection of 1933 participants, indicating any missing cases.
10. Clearly define the continuum of care, stating that 'Women who received all three stages of care (Full ANC, ID, and PNC) were considered to have undergone complete Continuum of Care.' Additionally, define Full ANC, ID, and PNC, and clarify how they were calculated, specifying whether women or live births or live births and still births were used as the denominator. Provide rationale for not including stillbirths, if applicable. Alternatively, consider mentioning this information in the sample size flow chart as suggested.
11. Why did not authors include essential variables for the current topic, such as "parity/ or birth order" and "distance to health facility," as covariates.
12. In the independent variables, "age" is mentioned, but in the table, it's labeled as "age at delivery." I suggest maintaining consistency. Additionally, the independent variables do not specify how media exposure, occupation, and health insurance were categorized.
13. Please review and confirm lines 141-143, and cross-check with the results output.
14. Given the smaller sample sizes for rural residence in Table 1, it's important to note that these smaller numbers should be interpreted cautiously to avoid misinterpretation of the results. Consider referencing how the DHS main report addresses and handles smaller sample sizes.
15. Check the spelling of "richer" in Table 2 and ensure that similar minor mistakes are corrected in other tables and categories as well.
16. Consider including "place of residence" in Table 2 and ensure its consistency throughout the table.
17. Consider visualizing the continuum of care for ANC, ANC+ID, and ANC+ID+PNC to enhance understanding.
18. Instead of simply writing "Wealth index combined (Table 2)," suggest using the term "wealth quintile" and providing a brief explanation in the methodology section, referring to the DHS wealth index calculation process.
19. Note that "Internet use" is included in Table 4 but not in Tables 1-3 and is also missing from the list of independent variables.
20. The first paragraph of the discussion should ideally be integrated into the introduction to provide background information for readers.
21. Include a discussion of maternal health inequalities in the introduction and link it to the disparities in continuum of care (CoC) discussed in the findings.
22. Maintain consistency in referring to NMICS or MICS.
23. Improve the discussion by linking evidence of maternal health service inequalities at both national and provincial levels. Consider examining supply-side data to provide further evidence of CoC disparities.
24. Ensure that when short forms like NMICS/MICS are used, they are explained or mentioned in parentheses upon first use to avoid confusion.
25. Overall comment: Check for grammatical errors and consider having the document proofread by native speakers for further refinement.
