# Supplementary material for: Continuum of care for maternal and newborn health services in Nepal: An analysis from demographic and health survey 2022
Source: PLoS One. 2025 Mar 24;20(3):e0319033. doi: 10.1371/journal.pone.0319033 (PMC11932462; doi:10.1371/journal.pone.0319033)
Supplement: Attachment — Submitted filename: Response to reviewers.docx [file pone.0319033.s003.docx]

**Reviewer 1**

Reviewer #1: Thank you for providing the opportunity to review the manuscript. The authors have conducted an analysis addressing critical issues, and addressing the gap in the continuum of care is essential for policymakers and program implementers. Once the below comments are addressed, I would recommend this manuscript for publication. However, I suggest reviewing the introduction and discussion sections with experts who have extensive knowledge about the current policies and programs related to maternal and neonatal health.

1. The authors mentioned logistic regression in the abstract, yet Poisson regression is detailed in the methodology section. Additionally, the findings present logistic regression output. Please review the report carefully to ensure coherence.

**Response:** Thank you for the feedback. We have corrected this part throughout the manuscript.

2. Be consistent in the use of decimal places, either using one or two after the decimal point throughout the document.

**Response:** Thank you. We have corrected data to two decimal place throughout the manuscript

3. Regarding the statement, "Madhesh residents had lower odds (AOR: 0.47, 95% CI: 0.23 - 0.99), Sudurpaschim had 27 higher odds (AOR: 2.37, 95% CI: 1.17 - 4.82) for ≥4 ANC visits and health facility delivery than Koshi," I question the significance of the upper limit of 0.99. If rounded to a single decimal place, it may not appear significant. Furthermore, please confirm if the associated p-value is less than 0.05.

**Response:** Thank you for the feedback. We have revised the regression model with additional variables in the model as suggested by reviewer and have opted caution while interpretation of findings with borderline significance.

It seems like the authors are trying to forcefully emphasize significance.

**Response:** Thank you for feedback. We have cautiously interpreted the findings with borderline significance and have also revised the discussion section.

4. I suggest adding 95% confidence intervals (CI) in the last sentence of the methods section in the abstract. The authors overlooked mentioning CI.

**Response:** We have corrected the write up.

5. In the introduction section of the main body, the authors discuss the SDG target for neonatal mortality. I suggest including a similar discussion for maternal mortality ratio.

**Response:** Thank you. We have revised the manuscript accordingly.

6. Consider including information about the policy scenario concerning maternal and neonatal health in Nepal in the introduction section. For example, mention initiatives like the Nepal Safe Motherhood and Newborn Health Road Map 2030 and elaborate on its focus on the continuum of care.

**Response:** Thank you. Revised accordingly.

7. It seems unnecessary for authors to use the abbreviation "MCVs" in parentheses (line # 73) since it's not used later in the text.

**Response:** Corrected in the text. We removed the abbreviation in parentheses

8. Please ensure the survey year is mentioned in the opening sentence of the methods section.

**Response:** Thank you. Corrected accordingly.

9. Simplify the discussion of sampling procedures in the methods section. Instead of detailed procedures, provide a brief overview and mention the total number of strata, refer readers to the main report for further details with citations. Additionally, consider including a flowchart illustrating the process leading to the selection of 1933 participants, indicating any missing cases.
10. Clearly define the continuum of care, stating that 'Women who received all three stages of care (Full ANC, ID, and PNC) were considered to have undergone complete Continuum of Care.' Additionally, define Full ANC, ID, and PNC, and clarify how they were calculated, specifying whether women or live births or live births and still births were used as the denominator. Provide rationale for not including stillbirths, if applicable. Alternatively, consider mentioning this information in the sample size flow chart as suggested.

Response: Thank you for feedback. Updated

11. Why did not authors include essential variables for the current topic, such as "parity/ or birth order" and "distance to health facility," as covariates.

**Response**: Thank you for feedback. We have revised the regression model adding the variables suggested and have revised the results and discussion section accordingly.

12. In the independent variables, "age" is mentioned, but in the table, it's labeled as "age at delivery." I suggest maintaining consistency. Additionally, the independent variables do not specify how media exposure, occupation, and health insurance were categorized.

**Response:** We have maintained consistency in tables and manuscript as suggested by reviewers. We have also added the operational definition of media exposure and health insurance.

13. Please review and confirm lines 141-143, and cross-check with the results output.

**Response:** Thank you for feedback. We have thoroughly revised the results section.

14. Given the smaller sample sizes for rural residence in Table 1, it's important to note that these smaller numbers should be interpreted cautiously to avoid misinterpretation of the results. **Consider referencing how the DHS main report addresses and handles smaller sample sizes.**

**Response**: Thank you for feedback. We have cautiously interpreted the findings considering smaller sample size in rural residence.

15. Check the spelling of "richer" in Table 2 and ensure that similar minor mistakes are corrected in other tables and categories as well.

**Response:** Corrected
16. Consider including "place of residence" in Table 2 and ensure its consistency throughout the table.

**Response:** Thank you for feedback. Corrected

17. Consider visualizing the continuum of care for ANC, ANC+ID, and ANC+ID+PNC to enhance understanding.

**Response:** Thank you for feedback. We have added Fig 1 that helps in visualizing ANC, ID, ANC +ID, PNC and ANC+ID+PNC.

18. Instead of simply writing "Wealth index combined (Table 2)," suggest using the term "wealth quintile" and providing a brief explanation in the methodology section, referring to the DHS wealth index calculation process.

**Response:** Thank you for feedback. We have revised the manuscript and have replaced wealth index combined with wealth quintile. Description has been added in methodology section, also referring DHS wealth index calculation.

19. Note that "Internet use" is included in Table 4 but not in Tables 1-3 and is also missing from the list of independent variables.

**Response:** Thank you for feedback. We have added Internet use in the list of independent variables

20. The first paragraph of the discussion should ideally be integrated into the introduction to provide background information for readers.

**Response:** We have revised the first paragraph of discussion with more closer linkage with introduction

21. Include a discussion of maternal health inequalities in the introduction and link it to the disparities in continuum of care (CoC) discussed in the findings.

**Response:** We have revised introduction section accordingly.

22. Maintain consistency in referring to NMICS or MICS.

**Response:** Thank you. We have replaced MICS with NMICS in all places.

23. Improve the discussion by linking evidence of maternal health service inequalities at both national and provincial levels. Consider examining supply-side data to provide further evidence of CoC disparities.

**Response:** Thank you. Discussion part has been revised

24. Ensure that when short forms like NMICS/MICS are used, they are explained or mentioned in parentheses upon first use to avoid confusion.

**Response:** We have expanded NMICS in first place it appears.

25. Overall comment: Check for grammatical errors and consider having the document proofread by native speakers for further refinement.

Thank you for feedback. We have thoroughly checked the manuscript for grammatical error and assume that it now meets the standards of PLOS ONE

**Reviewer 2**

**Feedback:** The findings can be important evidence for those who are involved in improving the maternal healthcare service utilization in Nepal. However, the authors lack adequate discussion of policy implication of their findings.

The AOR value for the wealthiest quintile seems unusually high (AOR: 11.96, 95% CI: 14.36 - 32.79). The reported AOR should fall within the range of the confidence interval resulting CI not aligning with AOR. Furthermore, AOR of ~12 indicates that individuals in the wealthiest/richest quintile have almost 12 times higher odds of receiving the combined maternal health services compared to the reference group. Although this is possible, it is unusually high and warrants further scrutiny considering the non-alignment of AOR and CI as mentioned above.

**Response:** Thank you for feedback. We have corrected findings in abstracts. We have revised the regression model as per feedback from reviewer 1 and findings now look more precise. We have checked consistency of findings reported throughout the manuscript.


**Feedback:** Stating the reference group is missing in some context.

**Response:** Thank you. We have revised.

**Feedback:** The conclusion is concise and straight forward. It could be further expanded slightly by linking with the policy implications or recommendations based on the findings which are more important for the readers. However, the authors have not discussed the policy implication in their discussion and obviously in the conclusion as well.

**Response:** Thank you for feedback. We have added policy implication in discussion and conclusion.


Introduction:

**Feedback:** Although the authors have mentioned a little about the importance of continuum of care, it would be useful to elaborate. This will allow readers the importance of such intervention that the authors are interested to explore further. Due to the lack of such information, the introduction section has limited theoretical underpinning for conducting this research. Why exploring the determinants of continuum of care is important?

**Response:** Thank you for the feedback. We have added a paragraph in introduction section addressing the feedback.

**Feedback:** The authors used the term Maternal Care Visits (MCVs) taking the reference #17. Is this term same with ‘Continuum of Care’ that the authors have used throughout this manuscript? What are the similarities and what are the differences of these terms?
**Response:** Thank you for feedback. We have maintained consistency in using continuum of care throughout the manuscript.


**Feedback:** Additionally, providing definitions for the variables of interest and explaining how the DHS formulated the survey questions (possibly in a separate table) would enhance the manuscript's clarity. This would ensure that readers understand, for example, that "PNC" refers specifically to "PNC visit for the mother within 2 days of delivery," as used throughout the manuscript.

Response: Thank you for feedback.


Method:

**Feedback:** The methodology section provides a thorough description of the sampling frame, data collection process, and statistical analysis. In data collection tools, the authors state that “DHS Program's model questionnaires were modified to address Nepal's unique demographics and health problems in consultation with various stakeholders, including government departments agencies, non-governmental organizations, and funders.” What were the unique demographics and health problems for Nepal identified after such consultation? What were such unique health problems that are relevant for this study? Were the respondents themselves also consulted before modifying the tools? How?

**Response:** The tool was revised considering the Nepalese context like ethnicity, classification of educational level, and type of facilities that are highly contextual. Revision was based on expert consultation.

**Feedback:** Clarify why only those women who had live births within two years preceding the survey were included in the analysis, and why this specific timeframe was chosen?

**Response:** This was to align with the standard approach in reporting maternal health service utilization so the findings are comparable. NDHS also reports indicators 2 years proceeding the survey.

**Feedback:** This manuscript does not mention how data cleaning was done. Since the authors used the DHS data source, DHS might have explained the data cleaning process elsewhere. A description of how DHS used any methods for cleaning the data would improve the robustness of the methodology.

**Response:** We used clean data from available from DHS site. Not to duplicate information, we have cited main report as needed in methodology part.


**Feedback:** The independent variables are listed but lack detailed definitions and categories in some places. Providing precise definitions (e.g. basic/secondary/higher education, urban/rural settings) and clarifying the dichotomization of religion variable (into Hindu and others, only in a religiously diverse society which could impact in health service utilization) would enhance further understanding.

**Response:** Thank you for feedback. Having long list of categories in independent variables may make regression model to perform weak, specially when numbers on specific categories are less. Recategorization and dichotomization has been an standard approach in running the regression models as in the previous literatures. Despite our best interest, we cannot have long list of categories for independent variables because of statistical reasons


Results:

**Feedback:** This section requires thorough consistency checks to avoid any similar discrepancies which have occurred in the abstract section earlier.

**Response:** Thank you for feedback. We have thoroughly revised the manuscript

**Feedback:** The overall number does not match with sum of urban and rural. Please check it.

**Response:** Findings section has been thoroughly updated including tables.

**Feedback:** I am not clear why the authors have done subgroup analysis in table 1. The rationale for conducting subgroup analysis might be required.

**Response:** We do not have subgroup analysis as such. However, data are presented in table 1 disaggregated by urban/rural setting. We assume providing detail data as possible could be informative from policy and programme perspective.

**Feedback:** I would suggest highlighting only key differences or changes the results to avoid repeating the same figures (line 154-157)

Response: Results section has been thoroughly revised

**Feedback:** Although the authors have numbered their table, this has not been mentioned in the text. I would suggest for citing table 1, 2, 3 ..... while writing the result section.

**Response:** Thank you. Table numbers are now quoted in text


Discussion:

**Feedback:** The authors argue that federal structure after the political change of 2015 and the transfer of authority to local level governments after the election of 2017 may have led to relatively better planning and effective implementation of interventions which increased the coverage in rural areas at a faster pace than urban areas in 2022 compared to 2016. Again, the authors have discussed the role of FCHVs in encouraging the utilization of MNCH services to increase the utilization in rural areas. Such arguments should be used cautiously. Does this mean, the FCHVs in urban areas (as per the definition of rural areas in DHS survey) have less contribution? To what extent this argument is valid? Is there any possibility that massive increase in number of municipalities/Urban areas after the political changes of 2017 could have resulted this variation? Is the definition of urban areas in 2016 comparable with the definition used in 2022?

**Response:** Thank you for feedback. The writeup was not meant to say FCHVs in rural setting work better than urban or have higher role. However, considering the high FCHV to population ratio, it is often not possible in urban setting to have complete engagement with every households with pregnant women. However, we have revised the manuscript to make sure that it does not communicate the wrong message.

We have updated the regression model as per feedback from reviewer 1. Urban/rural setting only have borderline association, which we have not emphasized in discussion as per feedback from reviewer 1. So, for this manuscript, whether recategorization of urban/rural setting has impacted the service utilization becomes irrelevant. However, that could be a matter for further studies.


**Feedback:** The authors stated that they found provincial variations in continuum of care for maternal health services utilization. The authors argue that management approaches and strategies adopted in expanding coverage within the provinces could be responsible for differences. What is the basis under this assumption? What does these management approaches mean here? How these approaches are different by provinces? Can this be clearly stated through the critical review of provincial health plans and policies (if exists)? Is the health plans and policies of Sudhurpaschim province has something different than other provinces which has resulted these findings? Are there specific policies or initiatives in Sudurpaschim that could serve as models for other provinces?

**Response:** thank you for feedback. We have now cautiously revised the discussion part


**Feedback** : Discussing about the results in discussion section, the manuscript lacks discussion on implications of wide confidence intervals in some cases. Wide CI in some instances suggest there might be significant variability within those subgroups analysis. The discussion of reasons behind such variability example [11.96 (4.36, 32.79) or even 21.15 (8.80, 50.86) in case of richest quintile] and their implication can enhance the manuscript quality.

**Response:** We have revised regression model and findings look more precise now.

**Feedback** One of the major drawbacks of this manuscript is it lacks the discussion on policy implication of their findings. For instance,


Age at delivery: I was interested to see the age at delivery for those who were below 20 years. Since the data was collected in 2022 and the survey asked for those who had live births within two years preceding the survey, what does it indicated with the existing laws of National Criminal Codes 2017 that criminalizes the acts of early marriage? How the health programs should be tailored to address this issue among the young population to prevent early marriage?

The use of internet services and health service utilization is interesting. What can be its policy implication? How can the IEC/BCC interventions be revamped considering the increased access to internet services over the period of time?

Government of Nepal has endorsed ANC to PNC Continnum of care Guideline <https://fwd.gov.np/wp-content/uploads/2023/06/ANC-to-PNC-continuum-of-care-guideline-4.pdf> . How can this findings be linked with the existing guidelines? What need to be added? These kinds of discussion might be required.

**Response:** Scope for improvement does not always mean we require policy changes. Implementation of already existing policies may also accelerate progress and we, based on this manuscript do not recommend any changes on ANC to PNC continuum of care guideline. The guideline is more related to components of care and largely a guiding document for health service providers.


**Feedback** These are only some examples. The discussion on separate subheading for policy implication is essential to make the manuscript better.

Response: Thank you. We have revised discussion with policy implication. However, we don’t have separate subheading in discussion complying with journal formatting and previous publications.

Conclusion:

As discussed in the abstract section, this conclusion is straight forward which needs further expansion by linking with the policy implications or recommendations based on the findings which are more important for the readers.

**Response:** Thank you for feedback. We have revised conclusion section both in abstract and main body of manuscript.
